# Exploring the Writing and Typing Needs of Older Adults with Acquired Vision Loss: A Qualitative Study of Challenges and Adaptive Strategies

**DOI:** 10.1007/s44402-026-00108-1

**Published:** 2026-06-10

**Authors:** Rukaiya Anwar, Jane Macnaughton, Keziah Latham

**Affiliations:** Vision and Hearing Sciences Research Centre, Anglia Ruskin University, Cambridge, UK

**Keywords:** Activities of daily living, Handwriting, Low vision rehabilitation, Psychosocial impact, Typing, Vision impairment

## Abstract

**Purpose:**

Writing is an essential yet often overlooked component of vision rehabilitation for adults with acquired vision loss, receiving less focus than activities such as reading. This study explores the specific challenges older adults encounter when engaging in writing tasks, the types of writing they wish to perform and the factors they associate with successful performance. It also examines the psychological and functional impacts of writing difficulties and evaluates the usability of current assistive tools designed to support writing.

**Methods:**

A qualitative design was employed using semi-structured interviews with 10 participants aged 67–93 years recruited through vision impairment support groups. Interview data were analysed using content analysis to identify key themes and subthemes related to participants’ needs, challenges and adaptive strategies.

**Results:**

Analysis identified five themes with 20 subthemes. Participants engaged in diverse writing tasks across daily, personal and occupational contexts, with emails, shopping lists and signatures being reported most frequently. Performance was limited by difficulties with initiation, multisensory coordination, visual focus and physical effort, but mitigated by task familiarity. Success was judged against personal benchmarks of spatial accuracy, legibility, efficiency and error correction, although these were often compromised. Adaptations included changes in modality, use of assistive technologies and reduction in frequency or volume of written output. Impact extended beyond function, contributing to frustration, embarrassment, reduced independence and social isolation.

**Conclusion:**

Participants emphasised the distinction between “spot” writing tasks, often retained for procedural familiarity and “fluent” tasks, requiring sustained effort. Success, from the patient perspective, was defined not by completion but by legibility, spatial accuracy, efficiency and self-correction, while preserving independence and identity. These findings highlight that outcome measures must reflect the lived experience of adults with acquired vision loss, capturing the functional, emotional and psychosocial dimensions of writing that matter most to people living with sight loss.

Key Points

- Differentiating between brief (spot) and sustained (fluent) writing tasks offers a new framework for understanding how varying writing demands are affected following acquired vision loss.
- Writing difficulties extend beyond task completion, affecting confidence, independence and social participation, highlighting the need for rehabilitation approaches that address both functional and emotional aspects of writing.
- Existing assessment approaches may overlook important aspects of writing performance, highlighting the need for outcome measures that reflect quality, effort and personal meaning in everyday written communication.

## Introduction

Writing has been identified as one of the top three vision rehabilitation priorities for adults with acquired vision loss, both at the initial assessment and 1 year later, highlighting its significance across the rehabilitation trajectory [1, 2]. Adults with acquired vision loss face distinct challenges that differ from those with congenital vision impairment (VI). Whereas individuals with congenital sight loss typically develop compensatory strategies from an early age through habilitation strategies [3], adults with acquired vision loss must relearn fundamental tasks, such as writing, through rehabilitation or self-directed adaptation later in life.

Despite its significance to communication, independence and self-expression, writing remains comparatively under-researched in the vision sciences and rehabilitation literature, particularly when contrasted with the emphasis placed on reading [4]. In this study, writing is defined as the physical production of text for communicative or functional purposes, and is considered across three modalities: handwriting, typing using a physical keyboard and typing using a touchscreen (virtual) keyboard.

Writing involves the integration of cognitive (idea generation, language formulation, executive control), motor (fine motor control for handwriting or typing) and visual (tracking, spatial orientation, error monitoring) elements, all coordinated to produce coherent written language across different modalities.

To some extent, writing is addressed within existing patient-reported outcome measures (PROMs), typically as individual task-based items rather than as part of a systematic assessment of writing ability. For example, writing-related tasks such as signing one’s name, completing forms or writing cheques appear frequently in PROM instruments [5–8]. The Participation Activity Inventory (PAI), for instance, includes the ability to fill out forms or write short notes, but these tasks are embedded within broader domains of communication tasks and not explored in depth [7]. The modality of such tasks is often unspecified (completing a form, for example, could involve either handwriting or typing), although certain items, such as signing or writing cheques, are exclusive to handwriting. Typing tasks are not explicitly represented within existing PROMs, despite their increasing relevance in everyday written communication.

A few experimental studies have examined specific aspects of the writing process in individuals with vision loss; for instance, difficulty in locating a pen tip in the presence of a central scotoma [9] or reduced typing accuracy associated with peripheral vision loss [10]. Although these studies provide valuable insights into particular challenges, the existing literature does not offer a comprehensive or integrative understanding of how individuals with acquired vision loss navigate writing tasks across different modalities, contexts and levels of complexity.

The aim of this study is to explore the lived experiences of adults with acquired vision loss in relation to writing qualitatively. Specifically, it investigates which writing tasks are considered important by participants, which elements are found most and least challenging, what adaptations and strategies are employed and how individuals define successful writing outcomes. These findings are intended to inform the development of a writing-specific outcome measure that reflects real-world priorities, preferences and experiences of people with VI, thereby contributing to more person-centred rehabilitation and assessment practices.

## Methods

### Participants

Participants were recruited through local sight loss charities based in Cambridge and Bristol (UK). Recruitment continued until the dataset was deemed sufficiently varied to enable to support in-depth analysis [11].

Participants were eligible for inclusion if they were adults (≥18 years of age) with self-reported, bilateral acquired vision loss that occurred after they had learned to read and write. Individuals were excluded if their vision loss resulted from neurological conditions likely to affect motor function (e.g., stroke) or if they were physically unable to write.

Participants were asked to report their VI registration status as part of a demographic questionnaire to provide an indication of the severity of sight loss. In the UK, individuals with permanent sight loss may be certified and registered as either Sight Impaired (SI) or Severely Sight Impaired (SSI), based on defined thresholds of distance visual acuity and/or visual field loss. These classifications follow criteria set out in the Royal College of Ophthalmologists (RCOphth) certification guidelines [12], which are widely used within UK ophthalmic and rehabilitation services to standardise definitions of sight loss. Therefore, collecting self-reported registration status enabled an overview of the range and severity of vision impairment within the sample.

The study was approved by the Faculty of Science and Engineering Research Ethics Panel at Anglia Ruskin University, UK (ETH2324-3052). Informed consent was obtained from all participants after passing the Six-Item Cognitive Impairment Screening Test [13].

### Interviews

Interviews were conducted by RA (an experienced optometrist) between May and July 2024, either in person or via video conferencing software (Microsoft Teams, Microsoft.com), depending on participant preference and accessibility. The interviews, lasting 60–90 min, were audio-recorded with participant consent. Transcriptions were created using Microsoft Teams and reviewed for accuracy before analysis, supported by NVivo 14 software (NVivo 14.24.0, QSR International; qsrinternational.com).

A semi-structured interview was used to address the following research questions:

1. What does the participant need and want to be able to handwrite or type?
2. What elements (e.g., cognitive, motor, visual) of writing (handwriting and typing) are most and least difficult?
3. What does success look like in a handwriting or typing task, and how is this measured?
4. What interventions are used or adaptations developed for writing? What are their benefits and limitations?

### Data Analysis

Data were analysed using content analysis to identify, categorise and interpret patterns systematically within the qualitative data [14, 15].

During the initial stage of content analysis, open coding was used by RA to review each transcript systematically and assign codes to segments of text relevant to the study’s research questions. The coding process was iterative and reflexive, allowing for ongoing refinement as new insights surfaced from the data [16]. An abductive approach was taken to the integration of codes into themes and sub-themes, combining both deductive and inductive reasoning to support a more dynamic analytical process [15, 17]. The abductive strategy enabled a comprehensive and grounded understanding of participants’ lived experiences, ensuring that findings were shaped by the realities of writing with acquired vision loss rather than being wholly constrained by predefined theoretical frameworks.

Following the initial identification of themes and sub-themes, the research team (RA, KL and JM), all of whom are qualified optometrists with experience in VI and rehabilitation services, engaged in a critical and reflective discussion to review the preliminary coding and thematic structure. Through a collaborative process, the team refined the categories by consolidating overlapping themes and clarifying category boundaries to ensure that all relevant data were represented accurately and that the resulting framework provided a comprehensive account of participants’ experiences.

In the final stage of analysis, the frequency with which each sub-theme was referenced was recorded as the number of participants who cited it. The resulting participant count (*n*) is used throughout the Results section to indicate the prevalence of specific experiences or viewpoints. All sub-themes are reported, regardless of frequency, to provide a comprehensive account of the range and variability of participants’ experiences [18].

## Results

Ten adult participants (five female, five male) aged 67–93 years (median age = 84 years) were recruited (Table 1).

**Table 1:** Participant (P) details, including sex, age, self-reported cause of vision loss, registration status and duration of vision impairment in years since diagnosis.

P number	Sex	Age (years)	Self-reported cause of vision loss	Registration status	Duration of vision impairment (years)
P1	Female	93	Wet AMD	SI	26
P2	Female	90	Dry AMD	SI	6
P3	Female	88	Wet AMD	SSI	7
P4	Female	87	Primary Open Angle Glaucoma	SI	9
P5	Male	85	Optic Neuropathy	SI	38
P6	Male	84	Wet AMD	SSI	2
P7	Male	83	Myopic Maculopathy	SI	21
P8	Female	63	Optic Neuropathy	SSI	12
P9	Male	74	Dry AMD	Not Registered	1
P10	Male	84	Glaucoma	Not Registered	6

*AMD* age-related macular degeneration, *SI* sight impaired, *SSI* severely sight impaired.

The data analysis yielded 81 primary codes, which were organised into five main themes and 20 subthemes. These themes reflect the key challenges and experiences reported by participants with regard to handwriting and typing following acquired vision loss. Four of the themes (1–4 in Table 2) were developed deductively based on the study’s research questions. An additional theme (number 5 in Table 2) was developed inductively from the data, reflecting unanticipated insights shared by participants that were not guided initially by the theoretical framework.

**Table 2:** Summary of themes and subthemes, listed in order from highest to lowest participant citation (*n* = 10).

	Themes	Sub-themes	Number of participants citing subtheme (*n*/10)
1	Writing task	1. Activity of daily living	10
		2. Personal	9
		3. Employment	4
2	Writing task performance	4. Task initiation	8
		5. Multisensory coordination	7
		6. Fatigue and maintaining visual focus	7
		7. Physical requirements	6
		8. Task familiarity	6
3	Writing task outcomes	9. Visuo-spatial accuracy	8
		10. Visual clarity	8
		11. Speed and efficiency	7
		12. Accuracy and correction	7
4	Writing intervention/adaptations	13. Change in writing modality	7
		14. Introduction of new device/tool	7
		15. Reduction of direct output	5
		16. Training	3
5	Psychosocial impact of sight loss on writing	17. Frustration	8
		18. Embarrassment	7
		19. Loss of Independence	7
		20. Social Isolation	6

Values indicate the number of participants who cited each subtheme.

### Writing Task

Tasks were categorised based on domains defined within the Occupational Therapy Practice Framework 4 (OTPF-4) [19], consolidated into three broad categories reflecting the primary contexts of occupational engagement: *Employment* (corresponding to the OTPF-4 domain of *Work*, including *Education* where applicable), *Personal* (encompassing aspects of Leisure and Social Participation) and Activities of Daily Living (ADLs) (including both *ADLs* and *Instrumental Activities of Daily Living (IADLs), and* incorporating *Health Management* where relevant). Domains such as *Rest and Sleep*, *Play* and *Education* were not represented as distinct categories, either because they did not appear in the data or because their content overlapped with broader areas of engagement (e.g., informal learning within the work context). Where writing tasks spanned multiple domains, such as managing medical forms that involved both personal communication and health management, they were categorised as *Shared* to reflect their intersection across occupational spheres.

**Fig. 1:**
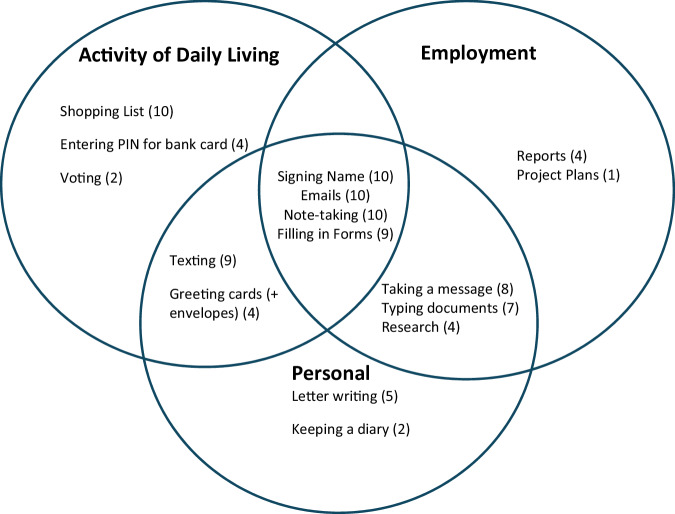
Handwriting and typing tasks as reported by participants living with acquired vision loss, categorised by domain. Numbers in brackets indicate the number of participants (out of 10) who mentioned each task. The figure includes all tasks reported as part of participants’ everyday lives, whether currently undertaken, previously performed or identified as personally important or challenging. All tasks mentioned during the interviews are included, regardless of frequency. PIN personal identification number.

Participants described a wide range of writing and typing tasks across everyday life (Fig. 1), spanning domestic, personal and occupational contexts. The most frequently cited tasks (emails, note-taking, shopping lists and signing one’s name) were each mentioned by all 10 participants. Other common tasks included filling in forms (9/10), texting (9/10) and taking messages (8/10), emphasising the importance of writing in both functional and social communication.

Three out of 10 participants described themselves as keen writers and expressed a strong desire to maintain this aspect of their lives. One participant shared:

*“I wrote quite a bit. I was a journalist before I had to retire. Writing was not just my job, but also a passion. I used to keep journals, write letters and of course, work on articles and reports. It was a big part of my life.” (P9)*.

Tasks varied in terms of modality. Some activities, such as entering a personal identification number (PIN) or composing emails, were typically completed through typing on a physical or virtual keyboard. Others, such as signing a name, required handwriting. Several tasks, including shopping lists, letters and forms, could be completed either via handwriting or typing, depending on the individual’s preference, ability and available tools. Where writing modality was interchangeable between handwriting and typing, it was often influenced by the form and complexity of the task at hand, with short-form tasks such as taking down a message being more likely to be completed with handwriting, and long-form tasks such as letters being typed.

*“I’ll handwrite short things because it’s easier in the moment. But for longer writing, I have to type, otherwise I lose track.” (P8)*

*“Shopping lists are okay because it’s one item at a time. I don’t have to keep track of a whole page.” (P2)*

*“If I only have to write a few words, I don’t think about it. If it’s a full page, I have to plan how I’m going to do it.” (P5)*

### Writing Task Performance

Task performance refers to participants’ experience of carrying out handwriting or typing activities, with a focus on the specific challenges encountered during the task, distinct from how they evaluated its outcomes. Participants described a range of physical, cognitive and sensory demands that influenced their ability to write following acquired vision loss.

The most common reported challenge was task initiation, described by eight out of 10 participants. Typically, writing requires preparatory actions such as gathering materials, setting up assistive tools and aligning paper or digital devices. For some, this stage was time-consuming and cognitively demanding, occasionally acting as a barrier to starting the task:

*“By the time I’ve got everything lined up, the paper, the light, the magnifier, I’ve almost talked myself out of it.” (P6)*

Seven participants described relying on multisensory input to compensate for reduced visual cues. Writing, whether typing or handwriting, often involves attention to sound, touch and kinaesthetic feedback. These adaptations were described as cognitively demanding, requiring participants to orient themselves through non-visual cues, such as the sound of keystrokes or the feel of a pen on paper:

*“I can’t see what I’m doing, so I’ve learned to feel my way through it: listen to the keys, feel the paper, just guess sometimes.” (P7)*

Maintaining visual focus during writing was reported by seven out of 10 participants and was closely associated with the experience of visual fatigue. Sustained concentration was required to monitor writing progress, especially when using magnification tools or screen readers. Participants described the physical strain of prolonged visual effort, often leading to tiredness and lapses in attention:

*“I have to concentrate so hard just to follow what I’m doing. After a few minutes, my eyes get tired and I lose track.” (P5)*

*“It’s exhausting, not just on my eyes but on my head - like I can’t keep going for long.” (P2)*

Six participants also described the physical demands of writing, particularly during longer tasks. These included hand fatigue, muscular discomfort and joint stiffness. Physical strain was frequently linked to compensatory postures and the effort required to maintain handwriting accuracy with reduced visual feedback:

*“I used to be able to write pages without thinking. Now, after just a few sentences, my hand feels sore and stiff. It’s like my body’s working harder to make up for what my eyes can’t do.” (P4)*

Task familiarity was reported by six participants as a factor that made certain writing activities more manageable. Repetitive, predictable or well-practised tasks, such as writing shopping lists or signing a name, were often completed with reduced reliance on visual monitoring. Participants described drawing on procedural memory to complete these tasks with greater ease:

*“I’ve written the same shopping list a hundred times. My hand remembers what to do, even if my eyes don’t.” (P5)*

These familiar tasks were typically short in duration and routine in nature. In contrast, long-form writing, such as emails, letters or extended notes, required greater effort across multiple domains, including task initiation, sensory coordination and physical endurance. Long-form writing tasks were more likely to result in fatigue or the need to switch to alternative input modes.

### Writing Task Outcome

In contrast to the process-related difficulties described earlier, participants also articulated a range of criteria by which they evaluated the success of completed writing tasks. These outcomes were concerned with the quality, usability and communicative effectiveness of the written product, rather than the cognitive, sensory or motor demands required to produce it. Four principal sub-themes were identified: visuo-spatial accuracy, visual clarity, speed and efficiency and accuracy and correction. These categories reflect the standards participants applied to their handwriting and typing and offer insight into the functional priorities and values that shaped their perceptions of success.

### Visuo-Spatial Accuracy

Visuo-Spatial accuracy included the ability to maintain precise spatial control over the placement of text when handwriting. Eight participants referred to challenges in writing within defined boundaries, such as ticking boxes on forms, signing on signature lines or maintaining a consistent horizontal trajectory across a page. Such tasks were significantly disrupted by reduced visual feedback, often resulting in misaligned, sloping or misplaced entries. For example:

*“Ticking a box or filling in a specific part of a form is really difficult: I’m always slightly off.” (P3)*

*“I can’t keep to a straight line. I start okay, but then it goes downhill, literally.” (P4)*

Where writing accuracy had procedural or legal consequences, these misplacements were especially anxiety-provoking. Participants viewed spatial control, not only as an aesthetic consideration, but as a practical and in some cases, institutional requirement.

### Visual Clarity

Visual clarity referred to the legibility, neatness and overall readability of the written output. The concept encompassed the clarity of letterforms, consistency of size and spacing and the extent to which the result was interpretable by others or by the participants themselves at a later time. Despite accurate placement of content, deterioration in visual precision frequently resulted in written work that was unreadable or unfit for use. For example:

*“I filled it [a form] in and then thought, God, I don’t think I can hand this in. She won’t be able to read it.” (P2)*

*“My letters are all over the place: some big, some small. It just doesn’t look right.” (P6)*

Clarity was also closely tied to personal standards. Eight participants expressed dissatisfaction with the appearance of their handwriting, noting that it no longer conveyed their intended message or sense of competence as a writer.

### Speed and Efficiency

The perceived success of writing tasks was influenced by the time and effort required to complete them. Seven participants described the writing process as markedly slower following vision loss, due to the need for deliberate pacing, frequent adjustments and reliance on assistive tools. The increased time demands affected not only productivity but also confidence in completing time-sensitive or public-facing tasks. For example:

*“Even writing a short note takes me three times as long. I have to go slowly to make sure it’s readable.” (P10)*

*“It depends on what the situation is, doesn’t it? If I’m trying to catch the post, then obviously there is time pressure.” (P1)*

While participants accepted a slower pace as part of adaptation, speed was still considered an indicator of success, particularly in contexts requiring real-time communication or where delays diminished the practical value of the written output.

### Accuracy and Correction

The final sub-theme concerned participants’ ability to avoid and amend errors during the handwriting and typing process. Mistakes, including misspellings, misaligned input or unintended keystrokes, were described by seven participants as frequent and difficult to detect without reliable visual monitoring. The corrective process itself was often described as laborious, requiring re-reading, magnification or auditory review via assistive technologies. For example:

*“I often must check what I’ve got on the screen and go, oh, there’s a wrong letter there. I’ve got displaced by one.” (P5)*

*“Success, for me, means being able to complete a handwriting task with minimal errors and frustration.” (P8)*

Notably, participants did not equate success with perfection, but with functionality. Writing tasks were deemed successful when they could be completed with a tolerable level of inaccuracy and minimal need for preparation and rework.

### Writing Interventions/Adaptations

Participants described a range of adaptations that supported handwriting and typing following acquired vision loss. Four overarching categories were identified: (1) change in writing modality, (2) introduction of new tools or devices, (3) reduction of direct output and (4) training or task familiarity. All participants reported using more than one strategy, and many described their approach to writing as a dynamic process of trial, adjustment and adaptation.

### Change in Writing Modality

Seven participants described shifting away from handwriting in favour of typing or voice-based input. These changes were often prompted by increasing difficulty with visual guidance, legibility or fatigue and were characterised as pragmatic responses to evolving functional capacity. Typing was widely preferred for longer tasks such as emails or documents, whereas handwriting was often reserved for brief notes or tasks where typing was impractical.

*“I’ve stopped trying to write by hand for anything long: email and messaging is just easier to manage now.” (P3)*

Some participants described using multiple modalities depending on task demands, context or environment. Such flexibility highlights the importance of preserving user autonomy in selecting communication methods that align with personal preference and individual task complexity.

### Introduction of New Tools or Devices

Seven participants reported incorporating assistive tools to support written communication (Table 3). These tools served various functions, including enhancing visibility, supporting spatial alignment, improving input accuracy and reducing cognitive effort. Devices ranged from digital technologies, such as large monitors and text-to-speech software, to low-tech adaptations, including bold-lined paper and tactile markers on keyboard keys. Most participants described these tools as helpful in enabling writing tasks across different contexts.

**Table 3:** Tools and devices adopted for writing by participants, highlighting reported positive and negative aspects and modality of application.

Tool/Device introduced	Reported positives	Reported negatives	Modality	Participants mentioned (*n*/10)
Screen magnifier	Helpful for long emails.	Requires training. Slows scanning.	Typing	6
Text-to-speech software	Provides auditory spelling feedback.	Hard to pause. Needs training for proper use.	Typing	5
Spellcheck	Quick and accessible.	Context errors. Not for major mistakes.	Typing	5
Bold-lined paper	Easier to see the lines.	May affect handwriting size and can be hard to find for purchase.	Handwriting	5
Hand magnifier	Makes everything bigger.	Results in only one other hand being available for holding the writing tool and or the writing pad/tablet.	Handwriting	5
Large print keyboard	Easier to see keys.	Covers more space. Increases errors.	Typing	4
Lighting	Better contrast overall.	May cause glare if not positioned correctly or used with electronic devices.	Both	4
Thick-tipped pen	Easier to see what has been written.	May be harder to read back what has been written and handwriting size may be affected.	Handwriting	4
Speech to text (dictation)	Enables text generation without handwriting or keyboard input; useful when physical writing is slow, effortful or inaccurate.	Reduced accuracy in noisy environments or due to regional accents; errors in speech recognition can disrupt writing flow; privacy concerns may limit use in public settings.	Both	4
Large screen	More magnified view	Takes longer to scan/navigate due to size.	Typing	3
Large stickers	Easier to see keys.	Can peel off easily.	Typing	3
Stylus	More precise positioning.	Need training for use.	Typing	2

Visual feedback tools, such as simple or digital magnifiers and large screens, were frequently used to improve clarity during extended writing. Audio-based supports, including read-back functions, were reported to assist with proofreading and reduce dependence on visual monitoring:

*“I use text-to-speech to check what I’ve typed; it picks up mistakes I’d otherwise miss.” (P6)*

These tools were most commonly used for longer or more cognitively demanding writing tasks, such as emails, forms or note-taking, where accuracy and legibility were prioritised. In contrast, they were used less often for brief or informal writing, where participants felt that the effort required to set up or access the technology outweighed the perceived benefit. Some participants also described selectively abandoning assistive tools in familiar environments, relying instead on habitual strategies or memory.

*“At home I don’t always use the tech. I know where everything is and I manage in my own way, so I just get on with it.” (P7)*

*“By the time I’ve opened the app and got it working, I could’ve just written the thing in my own way. For short bits, it’s not worth it.” (P5)*

The use of voice-based dictation was not included within the primary definition of physical writing, but was analysed as an adaptive strategy that participants adopted when traditional writing modalities became challenging.

Despite these benefits, most tools were associated with certain limitations. Several participants described difficulties in learning to use new devices, while others noted reduced writing speed or increased physical or cognitive effort:

*“It’s [iPad with assistive applications] another thing to carry around and you need good hand control to use it properly.” (P6)*

*“Typing is quicker and clearer, but it doesn’t feel the same; it’s not personal.” (P8)*

*“The voice app is convenient, but sometimes it messes up my words and that’s frustrating.” (P8)*

Participants also reported selecting different writing modes according to task type and context. Handwriting was often retained for socially or emotionally meaningful communication:

*“I still write birthday cards by hand - it shows I care.” (P10)*

In contrast, typing and voice input were preferred for practical, task-oriented writing:

*“Emails are just to get things done. If I want it to feel personal, I try to write it.” (P6)*

These findings suggest that whilst assistive tools were generally perceived as beneficial, their effective use depended on usability, familiarity and how well they aligned with individual preferences and routines.

### Reduction of Direct Output

Five participants reported adopting strategies to minimise the need for direct written output. These strategies included simplifying content, reducing the length of written tasks or delegating writing to others. While often effective in reducing task burden, such adaptations also introduced concerns around autonomy and self-expression.

*“It’s quicker when someone just helps me get it done.” (P6)*

*“You’re not independent. And if they’re not there, you’re stuck.” (P2)*

This theme reflects a functional compromise in which the efficiency gained through assistance or circumvention sometimes came at the cost of independence, confidence or privacy.

### Training and Task Familiarity

Three participants described the role of practice, routine and skill acquisition in improving their ability to manage written tasks. Rather than seeking to change the nature of the task or adopt a new tool, these individuals emphasised the value of becoming more familiar with existing strategies through repeated use.

*“You do get faster. It’s not perfect, but it’s about knowing the tool well enough to make it work for you.” (P4)*

This group also noted that routine tasks became easier over time, particularly those involving brief or predictable content. These short-form writing tasks required less visual focus and could be completed more automatically, in contrast to more cognitively demanding long-form writing tasks such as reports or emails.

### Psychosocial Impact

Participants described a range of consequences resulting from their reduced ability to write and type following acquired vision loss. Four sub-themes were identified: Frustration, Embarrassment, Loss of Independence and Social Isolation.

### Frustration

Frustration was the most reported emotional response, discussed by half of the participants. Handwriting and typing, once automatic and efficient, had become laborious and error-prone. Several participants described a sense of defeat, with one saying:

*“I’ve just completely given up on writing. I avoid it unless it’s absolutely necessary” (P10)*.

Others spoke about the repeated effort involved in making corrections, only to still be met with errors:

*“It’s a cycle. You write something, realise it’s wrong, try to fix it and then that’s wrong too. It just wears you down” (P5)*.

One participant recounted the experience of submitting a form with an incorrect tick box, stating:

*“They didn’t accept it because I made a mistake” (P3)*.

Assistive technologies were described as contributing to frustration, with complaints about screen readers being hard to control or too fast. These examples highlight how persistent difficulties led participants to reduce written output or disengage from writing altogether.

### Embarrassment

Embarrassment was raised by four participants and was largely linked to written communication in social or public settings:

*“It’s embarrassing when I must ask someone to check if I’ve signed in the right place. I used to be so independent.” (P4)*.

*“I was filling out a form at the post office and someone looked over my shoulder. I felt exposed and embarrassed” (P9)*.

These concerns also extended to personal gestures such as cards, where one participant shared:

*“I don’t like writing birthday cards anymore. I know it looks messy and I’m worried someone will think I didn’t make an effort” (P7)*.

### Loss of Independence

A loss of independence was reported by three participants, who reflected on their growing reliance on others for written tasks. The resulting shift was described as both inconvenient and emotionally challenging. One participant noted:

*“I used to be really independent, but now I rely a lot on others for help even for simple things like filling in a form” (P9)*.

In some cases, the inability to write independently led to the sharing of sensitive information. For example, one participant shared:

*“I had to give my PIN to my neighbour just so they could help me transfer money. It’s not ideal, but what choice do I have?” (P4)*.

Another described the emotional toll:

*“Every time I ask someone to write for me, it reminds me of what I can’t do anymore” (P6)*.

These accounts illustrate how writing challenges extended beyond inconvenience, undermining autonomy and personal privacy.

### Social Isolation

Three participants reported that reduced ability to write or type had contributed to feelings of social isolation. The decline in written communication, whether digital or handwritten, led to a weakening of social ties. One participant said:

*“I’ve missed communication with family. I used to write letters or messages. Now it feels like they’ve stopped expecting to hear from me” (P1)*.

Another discussed civic participation:

*“I couldn’t fill out forms or sign things anymore—even simple petitions or community surveys. It made me feel like my voice didn’t count, like I wasn’t part of things that mattered to everyone else.” (P2)*

A third participant described their limited interaction in a group chat:

*“I’m in a WhatsApp group with my friends, but I hardly message. I just read and react because typing takes so long” (P8)*.

These comments reveal how writing barriers can erode not only personal interaction but broader social engagement.

## Discussion

Older adults with acquired vision loss of varying duration and with conditions associated with both central and peripheral visual loss described varied challenges with handwriting and typing. These were captured across five key themes: *Writing Tasks, Writing Task Performance, Writing Task Outcomes, Writing Interventions/Adaptations and Psychosocial Impact*. Writing was portrayed as an enduring yet increasingly complex part of everyday life. Even when visual input was severely limited, it remained central to autonomy, identity and social participation. Participants emphasised its importance in maintaining daily routines, sustaining social connections and expressing personal identity. Losing the ability to write was experienced not as a minor inconvenience but as a disruption to role, competence and sense of self. Together, these themes highlight how writing extends beyond a visuo–motor act to encompass functional, cognitive, emotional and social dimensions.

### Writing Tasks: Scope, Purpose and Structure

A key conceptual contribution of the study lies in the distinction that emerged inductively during analysis between spot writing and fluent writing. This framing was developed to reflect participants’ consistent differentiation between short, discrete writing tasks and extended, continuous writing activities. For the purposes of analysis, *spot writing* is defined as brief, single-instance writing tasks requiring limited sustained visual monitoring or sequencing (e.g., writing a signature, short note or label), whereas *fluent writing* refers to extended writing tasks involving sustained sequencing, planning and ongoing monitoring of written output (e.g., composing emails, letters or reports). This mirrors conceptual frameworks in the reading literature, particularly the distinction between word and short phrase recognition and extended continuous text processing [20, 21]. Research on low vision reading has shown consistently that whilst many individuals retain the ability to decode short texts, continuous reading often becomes unsustainable due to visual fatigue, spatial disorientation and increased attentional load [22–24]. Findings from the current study suggest a comparable division in writing tasks.

Fluent writing involves extended or expressive writing tasks such as emails, letters or reports. Participants often described increased fatigue, frustration or disengagement when attempting fluent writing, particularly when using magnifiers or screen-based assistive tools. These findings parallel earlier findings showing that visual acuity alone does not account for reading performance in long-form contexts; endurance, attentional control and visual tracking become more influential over time [23, 24]. A similar pattern appears to underlie the contrast between spot and fluent writing: the issue is not simply one of legibility or motor function, but of cognitive load and task complexity.

While adaptations such as bold-lined paper or tactile guides supported spot writing, they were often insufficient for fluent writing. Transitions to typing or voice input were common, although not always seamless. Emotional meaning was often attached to fluent writing tasks, which were sometimes associated with communication, legacy or relational significance. Although the data did not indicate systematic emotional preferences for one writing mode over another, the continued use of handwriting in certain familiar contexts suggests that symbolic and personal factors may influence modality choice. Such findings align with previous literature recognising the enduring significance of handwriting as a tangible and identity-linked practice [25].

Recognising the distinction between spot and fluent writing could help inform more nuanced rehabilitation approaches. Spot writing may be supported through structured environments, tactile adaptations and reinforcement of procedural routines. Fluent writing may require strategies that reduce cognitive and visual load, including multimodal input, task segmentation and support with planning or editing processes. Interventions that take account of both functional and symbolic aspects of writing may offer more holistic support for people adjusting to vision loss.

Existing PROMs, such as the Activity Inventory [6], have provided essential tools for capturing vision-related function across multiple life domains, including communication. The present findings complement and build upon this work by suggesting that writing, as a subdomain of communication, encompasses distinct task types with differing demands. Incorporating a distinction between spot and fluent writing into future outcome measures may help to refine assessments of writing ability and better capture the diverse ways in which vision loss affects written communication.

### Task Performance

Writing challenges reported by participants extended beyond execution to include preparatory, sensory, motor and cognitive demands. Preparation itself emerged as a critical threshold: setting up paper, lighting or devices imposed considerable physical and mental effort. Several participants described this phase as discouraging, particularly when compounded by visual uncertainty, fatigue or anxiety about making errors.

To minimise the cognitive and visual demands associated with task preparation and setup, using structured environments may be useful. For example, maintaining a consistent and pre-configured workspace, with writing and typing tools positioned in fixed locations and digital accessibility settings preset, may reduce effort during task initiation.

Evidence from dual-task studies involving older adults suggests that combining sensory and cognitive demands increases perceived effort, and that environmental simplification can help mitigate this [26]. Preparation was especially effortful in fluent writing tasks, which required greater planning, setup and anticipated correction. Rather than viewing preparation as a separate preliminary step, it may be more appropriate to conceptualise it as an integral part of overall writing performance. Therefore, rehabilitation programmes could benefit from explicitly addressing preparatory demands as part of intervention design.

Participants frequently described using multisensory strategies to compensate for lost visual monitoring, including tactile feedback, auditory confirmation and procedural memory. These strategies often enabled continued writing, but came at a cost, particularly increased attentional load and fatigue during touchscreen use or magnified input. Similar trade-offs have been reported in experimental studies: audio-haptic cues can improve spatial accuracy and legibility, but tend to increase mental effort and task duration [10, 27, 28]. Older adults may be particularly vulnerable to such trade-offs due to reduced processing speed and working memory capacity [29]. Tools that streamline feedback, rather than layering multiple channels, may help reduce attentional switching and cognitive fatigue. Therefore, single-channel or simplified feedback mechanisms may be more suitable for sustained writing tasks, especially for fluent writing.

Physical strain was another consistently reported issue. Participants noted hand fatigue, awkward posture and musculoskeletal discomfort, particularly during prolonged or visually demanding writing. These experiences are consistent with research showing that visual feedback is essential for motor coordination and movement efficiency [9, 30]. Fatigue and strain increased with task duration and complexity: typically, spot writing tasks were manageable, whilst fluent writing was described as physically taxing. It might also be anticipated that fatigue and strain could be more pronounced for individuals relying on a preferred retinal locus or eccentric viewing strategies, although no participants mentioned this explicitly. Incorporating ergonomic strategies, such as optimised writing surfaces, adaptive grips, adjustable task lighting and seated posture alignment into rehabilitation may help reduce strain. These strategies could be supported by pacing recommendations and scheduled rest intervals to maintain performance over longer tasks. The occupational therapy literature has advocated similar approaches, particularly in interventions designed for progressive visual conditions [31].

Task familiarity appeared to mitigate both cognitive and physical demands. Participants reported that repetitive, well-practised tasks such as writing shopping lists, reminders or signatures could often be performed with minimal visual monitoring. Familiarity with the motor pattern allowed reliance on procedural memory, enhancing fluidity and confidence. These findings align with research in paediatric low vision rehabilitation, where structured repetition improved handwriting fluency and task independence [32, 33]. Embedding task-specific repetition and habit-building into interventions could strengthen procedural learning and reduce perceived effort during writing tasks. For instance, interventions could incorporate repeated practice of personally relevant writing tasks, such as completing shopping lists, medication logs or greeting cards, using consistent formats and routines to promote habit formation and reduce the effort required to initiate and complete the task.

### Task Outcomes: Redefining Success in Writing

Participants described successful writing as a multidimensional construct, encompassing more than task completion alone. Perceived success included dimensions such as spatial accuracy, legibility, fluency, efficiency and the ability to detect and correct errors. Some criteria were modality-specific, for example, physical alignment in handwriting and effective auditory feedback in typing, whilst others, such as visual neatness and fluency, were consistently valued across all forms of writing.

Visuo-spatial accuracy emerged as a key marker of success, valued not only for its practical implications but also for its psychosocial significance. Several participants expressed concern that poorly aligned or sloping handwriting might lead to formal documents being rejected or attract negative attention in public contexts. These accounts reflect broader evidence that vision impairment disrupts motor control and spatial consistency in handwriting [9, 30]. In this context, legibility and aesthetic presentation were often interpreted as indicators of personal dignity and competence, echoing findings that the visual quality of written output can influence confidence and self-perception among individuals with low vision [34, 35].

Efficiency also played a central role in shaping perceptions of success. While many participants tolerated slower writing speeds, particularly in personal or informal contexts, they described frustration when inefficiency compromised timely communication. Delays in responding to emails or completing written tasks led to feelings of exclusion or loss of control. These findings align with existing research showing that excessive inefficiency, particularly in the use of technology, can become a barrier to adoption and sustained engagement among older adults [10].

Participants also reported difficulty with error detection and correction, particularly in digital contexts where screen readers or auditory confirmation tools required sustained attention. Confirming the accuracy of written output often required multiple checks, which increased cognitive load and led to frustration. Experimental studies support these experiences: auditory scaffolding has been shown to improve error correction in 'blind' or vision impaired users but also imposes additional attentional demands [28].

Despite the significance participants placed on these nuanced aspects of writing, such dimensions remain underrepresented in existing outcome measures. Commonly used and newer PROMs, including the *PAI* [7], *National Eye Institute Visual Function Questionnaire–25 (NEI VFQ-25)* [5], *Impact of Vision Impairment (IVI) Questionnaire* [36] and the Low Vision Severely Constricted Peripheral Eyesight Questionnaire (LV SCOPE) [37], typically subsume writing under broad communication or daily living domains. In these instruments, writing is often reduced to a yes/no or scaled question regarding task difficulty (e.g., “How much difficulty do you have filling out forms?”), with little attention to performance quality, modality or psychosocial context. These measures capture whether individuals can complete writing tasks, but not how they do so or how satisfied they are with the result.

Where writing-specific tools exist, such as the *Low Vision Writing Assessment* [38], the evaluation is centred on performance across a small set of functional writing tasks, including writing a grocery list, completing a medical form, writing a paragraph of prose and managing cheque-based financial records. Participants may use any writing modality, provided the output is legible to an intended reader and tasks incorporate both spot and more fluent writing demands. The instrument uses an ordinal scoring system with a maximum raw score of 50, and while valuable in highlighting specific deficits in certain tasks, it is less clear whether the tool accounts for participant-defined success indicators such as efficiency, fluency and emotional resonance. These limitations may explain why writing-specific assessments have seen limited integration into routine practice, despite the recognised impact of writing on independence and self-expression.

### Adaptation Strategies: Tools, Modalities and Trade-offs

Participants described adaptation strategies as highly individualised, shaped by context, habit, task demands and available resources. Many reported transitioning from handwriting to digital modalities, such as typing or voice input, particularly for longer or more complex writing tasks. These shifts were described not as replacements for handwriting, but as practical responses to the physical, visual and cognitive demands of fluent writing. While these adaptations improved task completion and reduced reliance on visual feedback, they were rarely straightforward. Several participants encountered difficulties with unfamiliar interfaces, slower initial performance and physical discomfort, especially when using touchscreen devices or speech-to-text functions.

These experiences align with established findings on assistive technology use in older adults, where uptake is shaped by exposure, usability, contextual relevance and personalisation [39, 40]. In low vision populations, early-stage adoption is often hindered by ergonomic barriers, confidence loss and an increased cognitive load [34], as well as the availability of structured training and support [41].

Participants in this study emphasised that the provision of assistive tools alone was insufficient; developing effective use required hands-on support and task-specific guidance tailored to real-world writing needs. Recent research supports this approach: structured, skills-based instruction has been shown to improve proficiency and independence in assistive technology use significantly among older adults with VI [42, 43]. Training was not only instrumental in building confidence but also in helping participants view digital tools as viable methods, rather than emergency substitutes. These findings emphasise the importance of integrating sustained and practical training into rehabilitation programmes to support adaptation over time.

Assistive technologies such as screen readers, magnification and read-back functions were commonly used to support error detection, navigation and proofreading. Although participants acknowledged their usefulness, they also described cognitive strain due to attention-switching, sequential scanning and the mental effort required to track input across modalities. These challenges echo experimental findings that audio-visual substitution can increase cognitive load, particularly in older adults [28]. The effectiveness of such tools depended on appropriate configuration, task alignment and the user’s familiarity with the technology; support that was not always available consistently.

In some cases, participants adapted by reducing task complexity or seeking assistance from others. For instance, some chose to produce shorter written content, limit writing to essential tasks or ask family members to help with more complex correspondence. Although these strategies supported efficiency, they were occasionally experienced as limiting, especially when applied to private or sensitive tasks. Several participants expressed discomfort or frustration when adaptation resulted in reduced privacy or dependence. These findings echo previous studies suggesting that strategies promoting autonomy are perceived more positively than those increasing reliance on others [1, 35].

### Psychosocial Impact: Emotional, Relational and Participatory Effects

Writing difficulties following acquired vision loss had a profound psychosocial impact, extending beyond the technical challenges of handwriting or typing. Participants described emotional consequences that shaped their confidence, sense of agency and social engagement. These effects were cumulative rather than isolated and often intensified over time as routine tasks became more effortful or less reliably executed.

Frustration was one of the most reported responses. It was not limited to moments of error but emerged from the sustained effort required to complete tasks that had previously been automatic. Participants conveyed a sense of dissonance between their intent and the outcomes they were able to produce, mirroring broader findings that diminished performance in valued activities, which could erode motivation and self-efficacy [44]. Frustration was particularly acute in longer or more cognitively demanding writing tasks, where visual uncertainty, planning demands and fatigue combined to produce a sense of inefficiency and loss of fluency.

Embarrassment often accompanied visible evidence of impairment, such as misaligned handwriting, spelling mistakes or formatting errors in typed communication. Participants expressed concern about how others might interpret these irregularities, whether as signs of ageing, incompetence or reduced professionalism. In some cases, this led to self-censorship or avoidance of writing altogether, especially in contexts involving formal communication or emotionally significant exchanges. These reactions are consistent with research showing that perceived loss of control over appearance and output can affect confidence and contribute to social stigma [34, 35].

Loss of independence was particularly evident in participants’ reluctance to delegate writing tasks. While practical support from family members or carers was often necessary, it was not always welcomed. Tasks such as managing correspondence, completing forms or expressing personal messages were strongly associated with autonomy, privacy and competence. Requiring assistance in these areas could feel intrusive and was sometimes described as diminishing personal dignity. These findings align with previous work demonstrating that VI increases the risk of depression and emotional distress significantly, particularly when individuals experience a loss of control or reduced ability to manage everyday activities [45].

Social isolation emerged as a downstream effect of these experiences. Participants described withdrawing from written interactions, not due to lack of desire, but out of fear of making mistakes, falling behind or exposing their difficulties. Especially in digital environments, where communication tends to be fast-paced and accuracy is socially expected, some individuals disengaged altogether. These patterns reflect existing evidence that difficulties with communication technologies can reduce participation and increase loneliness among older adults with VI [10, 38].

Therefore, rehabilitation should consider psychosocial as well as technical outcomes. Importantly, this does not imply that separate psychological provision is always required; rather, well-designed practical interventions such as training that restores confidence or tools that safeguard privacy may themselves mitigate distress. For individuals with strong professional or expressive writing identities, opportunities for peer support or writing-focused rehabilitation may provide further benefit.

## Limitations

This study involved a small, relatively homogenous sample of 10 older British adults with acquired vision loss. While this sample size was appropriate for an in-depth qualitative design aimed at exploring lived experience [46], it necessarily restricts the diversity of perspectives represented.

Severity of VI was classified using participant self-report aligned with UK sight impairment (SI) and severe sight impairment (SSI) certification categories. Clinical measures of visual acuity or visual field function were not collected, limiting precise characterisation of impairment subtype (central/peripheral) or verification against formal criteria. There was greater representation of conditions associated with central VI, particularly age-related macular degeneration. As central and peripheral vision impairments are associated with differing functional demands during handwriting and typing, the themes identified may more strongly reflect experiences related to reduced central vision, such as difficulties with fine detail, letter formation and visual monitoring of written output. However, experiences linked to peripheral field loss, including challenges with spatial orientation, line tracking or locating materials within the workspace, were also evident and provide complementary perspectives on writing with VI.

The cross-sectional design reflects participants’ experiences at a single point in time and does not capture how writing practices and coping strategies may change in response to disease progression, technological developments or shifts in personal circumstances. However, individuals with vision loss of varying duration (1–38 years, Table 1) were included.

While the study focused on handwriting and typing as the primary modes of written communication, the rapid evolution of assistive technologies may affect the long-term relevance of tool-specific insights. However, the broader themes identified remain applicable to understanding the experience of writing with VI. Additionally, all researchers were optometrists, resulting in an optometric perspective in the analysis. However, findings are intended to be relevant to a multidisciplinary audience in low vision research and rehabilitation.

## Conclusion

Writing remains central to autonomy, identity and social participation for older adults with acquired vision loss, yet it presents complex and multifaceted challenges. Participants’ accounts demonstrated that writing is not solely a functional task, but a practice imbued with emotional, symbolic and personal significance. The distinction between short-form and long-form writing tasks, conceptualised as spot and fluent writing, highlights the wide spectrum of motor, cognitive and psychosocial demands. Difficulties across handwriting, typing and speech-to-text modalities point to the need for rehabilitation approaches that are both individualised and adaptable.

Recognising writing as a distinct and meaningful domain of rehabilitation, rather than a subsidiary element of communication or daily living, is critical. Interventions should be tailored to task type and user preference, incorporating structured practice, procedural reinforcement, strategies for error management and environmental adaptation. Addressing the emotional consequences of writing loss is equally important. Experiences of frustration, embarrassment and withdrawal were common, but could be mitigated through confidence-building approaches, privacy-preserving technologies and peer-led support that foster a sense of control and motivation.

There is a clear need to develop a writing-specific outcome framework that reflects both performance and lived experience. Existing instruments often assess task completion without capturing dimensions such as spatial accuracy, legibility, efficiency, error correction or emotional impact. A user-informed measure that accommodates different modalities and writing demands would better align evaluation with the realities of adapting to vision loss.

Overall, the findings support a model of rehabilitation that integrates technical skill development with psychological and identity-based support. Writing should be understood not merely as a mode of communication, but as a vehicle for preserving dignity, autonomy and connection. Embedding this understanding into both evaluation and intervention can enhance the relevance and impact of rehabilitation, supporting older adults to sustain meaningful engagement in their daily lives through writing.
